# Unraveling the Nephroprotective Potential of Papaverine against Cisplatin Toxicity through Mitigating Oxidative Stress and Inflammation: Insights from In Silico, In Vitro, and In Vivo Investigations

**DOI:** 10.3390/molecules29091927

**Published:** 2024-04-23

**Authors:** Shimaa A. Abass, Abdullah A. Elgazar, Sanad S. El-kholy, Amal I. El-Refaiy, Reem A. Nawaya, Mashooq Ahmad Bhat, Foad A. Farrag, Abdelrahman Hamdi, Marwa Balaha, Mohammed A. El-Magd

**Affiliations:** 1 Department of Biochemistry, Faculty of Pharmacy, Kafrelsheikh University, Kafrelsheikh 33516, Egypt; remapharma2010@yahoo.com; 2 Department of Pharmacognosy, Faculty of Pharmacy, Kafrelsheikh University, Kafrelsheikh 33516, Egypt; abdulah.elgazar@phr.mans.edu.eg; 3 Department of Physiology, Faculty of Medicine, Kafrelsheikh University, Kafrelsheikh 33516, Egypt; sanad_elkholy2014@med.kfs.edu.eg; 4 Department of Agricultural Zoology and Nematology, Faculty of Agriculture (Girls), Al-Azhar University, Cairo 11884, Egypt; amal.ibrahim@azhar.edu.eg; 5 Department of Pharmaceutical Chemistry, College of Pharmacy, King Saud University, Riyadh 11451, Saudi Arabia; mabhat@ksu.edu.sa; 6 Department of Anatomy, Faculty of Veterinary Medicine, Kafrelsheikh University, Kafrelsheikh 33516, Egypt; foad.farrag@vet.kfs.edu.eg; 7 Department of Pharmaceutical Organic Chemistry, Faculty of Pharmacy, Mansoura University, Mansoura 35516, Egypt; abdelrahmanhamdi2012@yahoo.com; 8 Department of Medical, Oral and Biotechnological Sciences, “G. d’Annunzio” University of Chieti-Pescara, Via dei vestini, 31-66100 Chieti, Italy; marwa.balaha@unich.it

**Keywords:** drug repurposing papaverine, cisplatin, nephrotoxicity, inflammation, network pharmacology

## Abstract

Cisplatin is a potent compound in anti-tumor chemotherapy; however, its clinical utility is hampered by dose-limiting nephrotoxicity. This study investigated whether papaverine could mitigate cisplatin-induced kidney damage while preserving its chemotherapeutic efficacy. Integrative bioinformatics analysis predicted papaverine modulation of the mechanistic pathways related to cisplatin renal toxicity; notably, mitogen-activated protein kinase 1 (MAPK1) signaling. We validated protective effects in normal kidney cells without interfering with cisplatin cytotoxicity on a cancer cell line. Concurrent in vivo administration of papaverine alongside cisplatin in rats prevented elevations in nephrotoxicity markers, including serum creatinine, blood urea nitrogen, and renal oxidative stress markers (malondialdehyde, inducible nitric oxide synthase (iNOS), and pro-inflammatory cytokines), as tumor necrosis factor alpha (TNF-α), monocyte chemoattractant protein 1 (MCP-1), and interleukin-6 (IL-6). Papaverine also reduced apoptosis markers such as Bcl2 and Bcl-2–associated X protein (Bax) and kidney injury molecule-1 (KIM-1), and histological damage. In addition, it upregulates antioxidant enzymes like catalase (CAT), superoxide dismutase (SOD) and glutathione peroxidase (GPx) while boosting anti-inflammatory signaling interleukin-10 (IL-10). These effects were underlined by the ability of Papaverine to downregulate MAPK-1 expression. Overall, these findings show papaverine could protect against cisplatin kidney damage without reducing its cytotoxic activity. Further research would allow the transition of these results to clinical practice.

## 1. Introduction

Cisplatin (CP), an inorganic platinum derivative, is a chemotherapy compound commonly used to treat different solid tumors. CP induces DNA adducts and DNA crosslinks, initiates DNA degradation, and disrupts the cell cycle, all of which lead to the death of cancer cells [1]. Unfortunately, severe adverse effects are reported in patients receiving CP, including nephrotoxicity, which is considered the major side effect of CP administration [2]. The risk of nephrotoxicity of CP ranges from 20% to 35%, which may lead to death in patients with acute kidney injury [3]. Various cytotoxic mechanisms are involved in CP nephrotoxicity. In addition to DNA damage, CP also induces the dysfunction of the cytoplasmic organelle, especially in the mitochondria and endoplasmic reticulum, leading to the activation of apoptotic pathways and causing cellular damage through inflammation and oxidative stress [4]. It is believed that inflammatory responses, particularly those involving the nuclear factor-κB (NF-κB) signaling pathway, are the main cause of CP-mediated renal damage [5]. Moreover, it has been reported that MAPKs play a pivotal role in the induction of CP-induced toxicity [4,6].

Despite the clinically limiting nephrotoxicity induced by cisplatin, there are currently no effective drugs used to prevent this side effect. Treatment safety is considered an important issue regarding studies related to human subjects. The ideal protective agent should be effective at lower doses, nontoxic, economical, and easily available [7]. Recently, research has been conducted on natural agents due to their ability to reduce cancer incidence and antioxidant activity and suppress drug-associated toxicities [8]. Due to the multitherapeutic activity of natural products, they could act as potential agents for suppressing oxidative stress-related cellular pathologies, including nephrotoxicity caused by CP administration [9,10]. Hence, many natural products have been investigated for their potential to protect kidney function by restoring the redox balance disrupted by cisplatin [11].

It was reported that the nitric oxide (NO) and the cyclic 3′,5′ guanosine monophosphate (cGMP) axis play a crucial role in maintaining renal perfusion and glomerular filtration [6]. Endothelial NO synthase (eNOS) in vascular endothelial cells converts L-arginine into NO and exerts many of its activities through the activation of cGMP, whose level is controlled by phosphodiesterase (PDE) [12]. Renal injury was shown in response to PDE activation, whereas renoprotection was seen in response to PDE inhibition [13]. PDE inhibitors also have anti-inflammatory effects [14]. Papaverine (PV), a benzylisoquinoline non-narcotic opium alkaloid extracted from *Papaver somniferum*, is a selective PDE10 inhibitor that also has anti-inflammatory and antioxidant properties and is clinically used as a vasodilator [15,16].

The pleotropic nature of drug-induced nephrotoxicity requires the regulation of several molecular pathways. Hence, it is logical to utilize pharmacological agents with multitherapeutic activities. As previously established, PV showed a remarkable ability to modulate several genes involved in inflammation, oxidative stress, and apoptosis. However, its role in nephrotoxicity has not been clearly illustrated yet.

Network pharmacology has recently gained traction as a tool to unravel mechanisms underlying natural compounds’ bioactivity profiles and diverse health effects. This computational approach enables predictions beyond conventional bioassays, thus fostering new therapeutic applications to manage complex chronic diseases [17,18].

Therefore, in this study, we utilized network pharmacology to illuminate pathways linked to nephrotoxicity that could be modulated by papaverine (PV). We then validated key predictions in vitro by evaluating PV’s effects on normal and cancer cell proliferation. Finally, we assessed PV’s ability to alter gene expressions underlying these nephrotoxic mechanisms in vivo using a rat model, alongside histopathological and functional outcomes, to confirm the compound’s protective effects against cisplatin-induced kidney damage.

## 2. Results

### 2.1. Network Pharmacology and Molecular Docking Analysis

**Figure 1:**
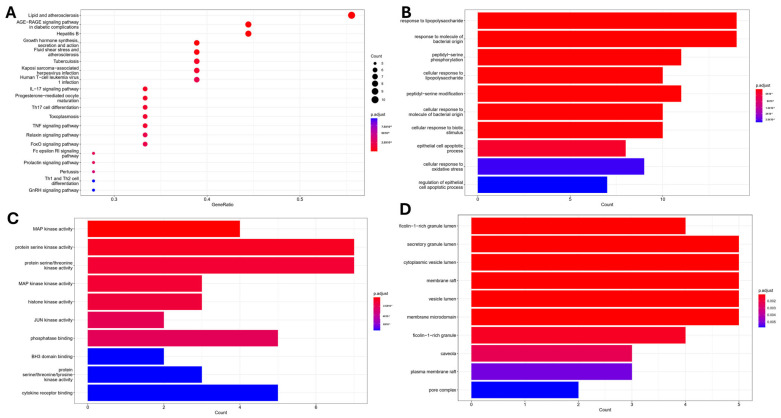
Enrichment analysis of terms related to cisplatin nephrotoxicity and predicted targets associated with potential papaverine activity: (**A**) KEGG; (**B**) BP; (**C**) MF; (**D**) CC.

Target fishing predictions identified twenty protein targets potentially involved in the nephroprotective effects of papaverine (PV). Enrichment analysis of these targets using Gene Ontology (GO) and Kyoto Encyclopedia of Genes and Genomes (KEGG) pathway mappings revealed associated biological processes (BP), molecular functions (MF), and cellular components (CC) underlying PV’s mechanisms of action against kidney injury. Interestingly, top-enriched BP were related to lipopolysaccharide inflammation, the response to molecules of bacterial origin, and peptidyl-serine phosphorylation (Figure 1A). MAP Kinase activity and protein serine/threonine kinase activity were highlighted as the most important MF (Figure 1B), while membrane raft, secretory granule lumen, and cytoplasmic vesicle lumen were identified as the most enriched terms for CC (Figure 1C). KEGG-enriched terms were associated with pathways related to the AGE-RAGE signaling pathway, lipid, and atherosclerosis, as well as IL-17 and TNFα signaling pathway (Figure 1D), indicating that PV exerts its activity through modulation of oxidative stress and inflammatory pathway.

**Figure 2:**
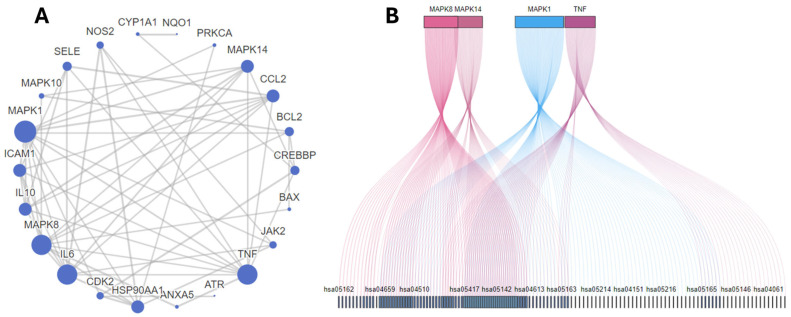
(**A**) Target proteins expected to be involved in PV activity and their interactions with one another. (**B**) The top five targets included in KEGG pathways, with more overlap between genes indicating increased involvement of shared pathways. Connections between genes and pathways are shown by lines of varying colors.

To obtain further explanation about other pathways associated with the nephroprotective effect of PV, a protein–protein interaction network was constructed where interactions were filtered based on a confidence score threshold of 0.7 to ensure reliable interactions in our network. MAPK1, 8, 10, and 14 were responsible for most of the interactions. Moreover, HSP90AA, CDK2, JAK2, CREBBP, ICAM1, and NOS were highlighted among the top 10 targets (Table 1, Figure 2A,B). MAPK1 was shown to be the most significant target as it was involved in the interaction with 100 out of 144 targets enriched from the KEGG, indicating its importance as the main pathway for PV to exert its action.

**Figure 3:**
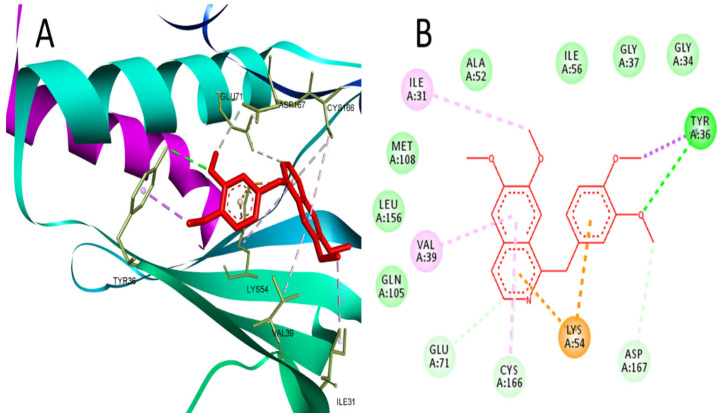
(**A**) 3D interaction of PV in the binding site of MAPK1 (PDB:4O6E). (**B**) 2D interaction of PV in the binding site of MAPK1.

Further confirmation for targeting MAPK1 by PV was performed using docking, and the results revealed the ability of PV to bind MAPK1 at the same active site of the co-crystallized ligand (2SH), which is a pyrimidine-based inhibitor of MAPK1. At the binding site of MAPK1, PV interacted with Tyr-36 through hydrogen bonding and Ile31, Val39, Lys54, Glu71, Cys166, and Asp167 through hydrophobic interactions (Figure 3). PV achieved a reasonable binding energy (−24.5) in comparison to the co-crystallized ligand (−30.5).

### 2.2. Effect of PV on CP-Induced Cytotoxicity on Cancer and Normal Cells

**Figure 4:**
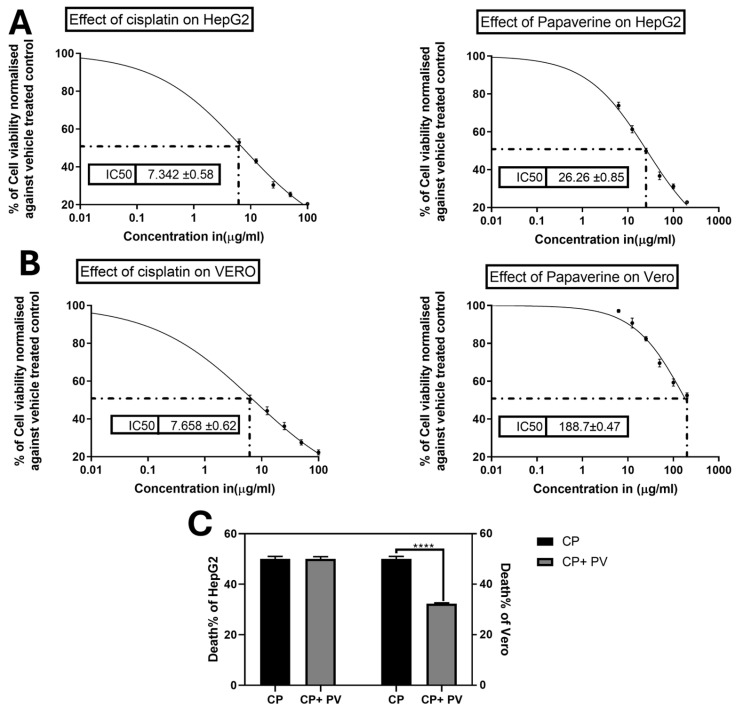
Effects of cisplatin (CP) and papaverine (PV) on the viability of liver cancer HepG2 and normal kidney Vero cells as measured by the MTT assay (**A**) IC50 of CP and PV on HepG2 (**B**) IC50 of CP and PV on Vero. (**C**) Effect of combination of PV (10 µg/mL) with CP IC50 on HepG2 and Vero viability in comparison to cells treated with CP only. Data are expressed as mean ± SEM, *n* = 3. **** *p* < 0.0001.

CP is known for its notorious cytotoxicity on normal cells since it has a low selectivity index. In order to investigate the ability of PV to protect against such toxicity without halting the CP effect on cancer cells, the MTT assay was conducted to determine IC_50_ of CP and/or PV on liver cancer HepG2 and normal kidney Vero cells. CP showed lower IC_50_ values of 7.3 and 7.658 µg/mL, while PV exhibited higher IC_50_ values of 26.26 and 188.7 µg/mL on Hepg2 and Vero cells, respectively (Figure 4). Additionally, the cytoprotective effect of PV against CP was assessed by concomitant treatment of the cells at the IC_50_ of CP and a subtoxic dose of PV (10 µg/mL) to ensure that PV would not interfere with the proliferation of Vero or HepG2 cells. Interestingly, PV was able to reduce the cytotoxic effect of CP on Vero by 35% in comparison to cells treated with CP alone. Remarkably, combined treatment of CP with PV did not affect its ability to inhibit the proliferation of HepG2 cells, implying the ability of PV to substantially increase the selectivity index of CP (Figure 4).

### 2.3. PV Decreased Serum Levels of CP-Triggered Renal Damage Parameters

**Figure 5:**
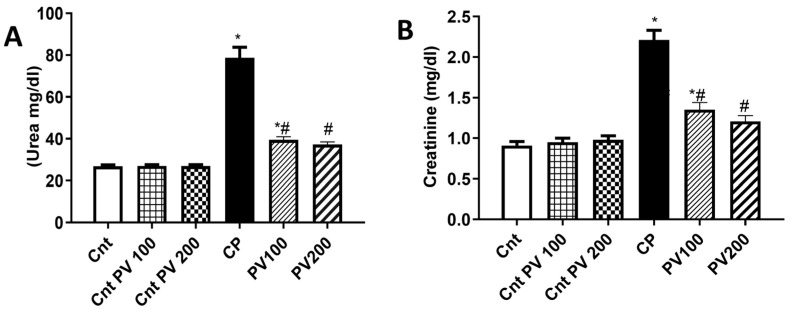
Serum level of (**A**) urea and (**B**) creatinine in different rat groups. Data were expressed as mean ± SEM (*n* = 7). * Moreover, # represents a significant difference at *p* < 0.05 against the control (Cnt) and cisplatin (CP) groups. PV100, low dose papaverine-treated group; PV200, high dose papaverine-treated group.

As illustrated in Figure 5, CP-treated rats showed significantly (*p* < 0.05) higher serum urea and creatinine levels than the three control (Cnt, Cnt PV 100, and Cnt PV 200) groups. The treatment with PV 100 mg/kg, or 200 mg/kg, showed a significant (*p* < 0.05) reduction in the serum levels of urea and creatinine, with the lowest level in the PV 200 group, compared to the CP group. Unfortunately, none of the treated groups improved to the level of the controls. Furthermore, there were no statistically significant differences between the three control groups.

### 2.4. PV Diminished CP-Promoted Renal Oxidative Stress

**Figure 6:**
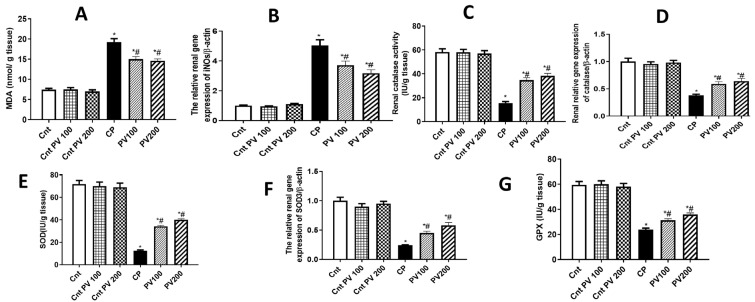
Treatment with PV protected against CP-induced renal oxidative stress as revealed by oxidative stress parameters ((**A**) MDA levels and (**B**) iNOs expression) and activities of the antioxidant enzymes ((**C**) CAT, (**E**) SOD, and (**G**) GPx) and their related genes ((**D**) CAT and (**F**) SOD3). Data were expressed as mean ± SEM (*n* = 7). * Moreover, # represents a significant difference at *p* < 0.05 against the control (Cnt) and cisplatin (CP) groups. PV100, low dose papaverine-treated group; PV200, high dose papaverine-treated group.

It is commonly believed that the molecular imbalance of oxidative stress contributes to the progression and development of acute and chronic kidney diseases [19,20,21]. Administration of CP could promote a significant (*p* < 0.05) increase in MDA levels and a significant (*p* < 0.05) decrease in the antioxidant capacity as revealed by a decline in the renal activities of CAT, SOD, and GPx, relative to the three control groups (Figure 6). However, the treatment with 100 mg or 200 mg PV showed significantly (*p* < 0.05) decreased MDA levels and significantly (*p* < 0.05) increased antioxidant enzyme activities when compared to the CP group (Figure 6). The antioxidant potential of PV was confirmed at a molecular level using qPCR, and the results showed significantly (*p* < 0.05) higher renal expression of the oxidative gene iNOs and significantly (*p* < 0.05) lower expression of the antioxidant SOD3 and CAT genes in the CP group than in the three control groups (Figure 6). Whereas the treatment with PV 100 mg or 200 mg significantly (*p* < 0.05) downregulated iNOs and significantly upregulated SOD3 and CAT gene expression, with better effects for PV 200 mg, relative to the CP group. Our findings indicate that PV could attenuate renal oxidative stress induced by CP.

### 2.5. PV Inhibited Renal Inflammation Caused by CP

**Figure 7:**
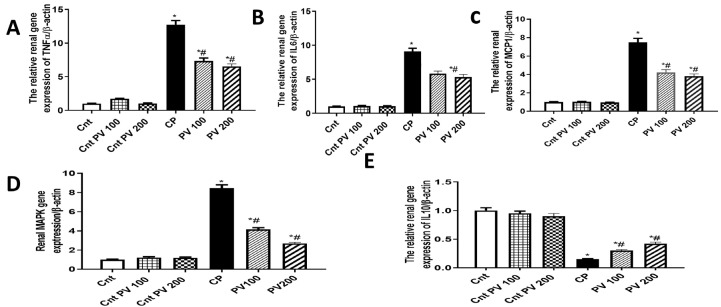
The impact of PV and/or CP on the expression of inflammation-related genes ((**A**) TNFα, (**B**) IL6, (**C**) MCP1, (**D**) MAPK1, (**E**) IL10) in kidney tissues as determined by qPCR. Data were expressed as mean ± SEM (*n* = 5). * Moreover, # represents a significant difference at *p* < 0.05 against the control (Cnt) and cisplatin (CP) groups. PV100, low dose papaverine-treated group; PV200, high dose papaverine-treated group.

The renal expression of the inflammatory genes TNFα, IL6, MCP1, and MAPK1 in the CP group was significantly (*p* < 0.05) increased when compared to the three control groups (Figure 7). In contrast, the anti-inflammatory IL10 gene was significantly (*p* < 0.05) decreased in the CP group compared to the three control groups. However, the treatment with PV 100 mg or 200 mg significantly (*p* < 0.05) reduced the four measured inflammatory genes and significantly (*p* < 0.05) upregulated the expression of the anti-inflammatory IL10 in renal tissues, compared to the CP group (Figure 7).

### 2.6. PV Alleviated CP-Induced Renal Apoptosis

**Figure 8:**
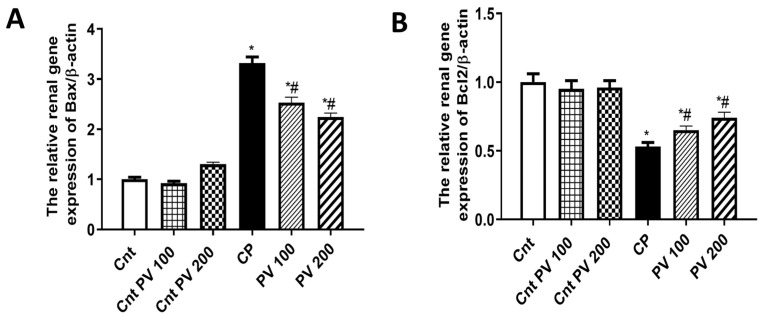
The effect of PV and CP on the renal expression of (**A**) the apoptotic gene (Bax) and (**B**) the anti-apoptotic gene (Bcl2) as detected by qPCR. Data were expressed as mean ± SEM (*n* = 7). * Moreover, # represents a significant difference at *p* < 0.05 against the control (Cnt) and cisplatin (CP) groups. PV100, low dose papaverine-treated group; PV200, high dose papaverine-treated group.

Apoptosis is an important molecular pathway involved in CP-induced nephrotoxicity [22]. B-cell lymphoma protein 2 (Bcl-2)-associated X (Bax) and Bcl-2 are two cytoplasmic proteins that serve as a promoter and an inhibitor of apoptosis, respectively [23]. Consequently, we assessed the impact of PV on CP-induced renal cell apoptosis by measuring the relative expression of the apoptotic Bax and the anti-apoptotic Bcl2 genes. The CP group showed a significant (*p* < 0.05) elevation in Bax and a significant (*p* < 0.05) decrease in Bcl2 compared to the three control groups. However, the treatment with PV100 mg or 200 mg exhibited a significant (*p* < 0.05) downregulated expression of Bax and a significant (*p* < 0.05) upregulation of Bcl2 relative to the CP group (Figure 8). These findings supported the anti-apoptotic role of PV against CP-mediated nephrotoxicity.

### 2.7. PV Restored Renal Damage Cause by CP

**Figure 9:**
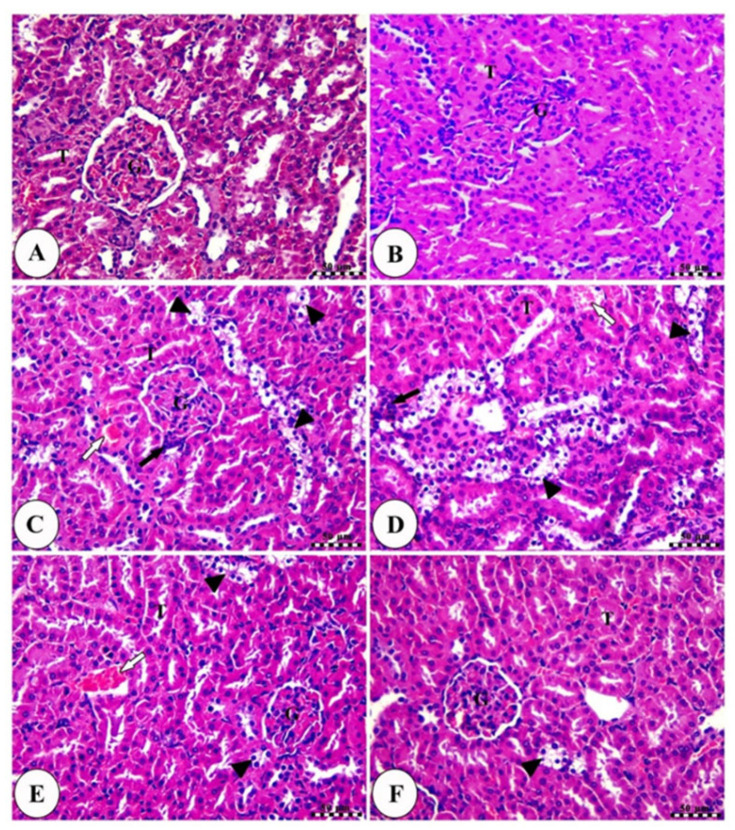

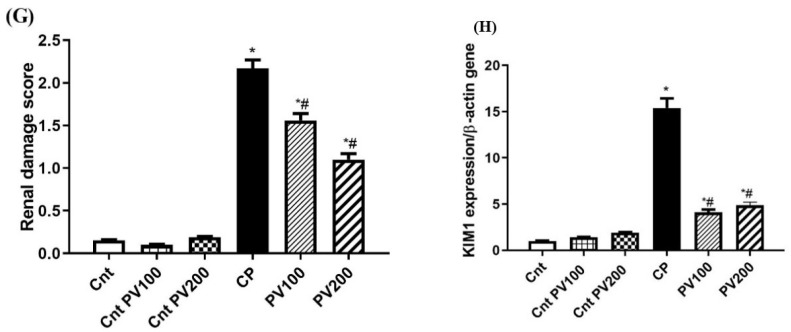
Photomicrographs of kidney sections in the normal control group (**A**), the control PV200 group (**B**), the CP group (**C**,**D**), the PV100-treated group (**E**), and the PV200-treated group (**F**). All slides were stained with H&E, scale bars = 50 µm. All labels (arrows and arrowheads) were explained in the main text. (**G**) Renal damage score. (**H**) The effect of PV on KIM1 gene expression. Data were presented as mean ± SEM. * Moreover, # represents a significant difference against the control and cisplatin groups. Cnt: Control, CP: cisplatin, PV: papaverine.

The histopathological examinations of the normal (healthy) and PV200 control groups showed normal renal parenchyma with renal corpuscles containing intact renal glomeruli surrounded by narrow glomerular space and bounded by intact glomerular capsule in addition to intact proximal and distal convoluted tubules (Figure 9A,B). In contrast, the CP-treated group revealed severe vacuolar degeneration of renal tubules with nuclear pyknosis in addition to moderate congestion of renal blood vessels (Figure 9C,D). On the other hand, low and high doses of PV-treated groups exhibited significant amelioration of renal tubules and a mild degree of congestion of renal blood vessels, especially with the best effect for the higher dose (Figure 9E,F). The kidney damage score showed that the CP group had significantly (*p* < 0.05) more renal damage than the other groups (Figure 9G). This score was significantly (*p* < 0.05) lower in the treatment groups compared to the CP group, with the PV 200 group having the lowest score.

### 2.8. PV Repressed CP-Induced Renal Damage

KIM1, a type 1 transmembrane protein, has mucin and an immunoglobulin domain, whose expression is significantly upregulated in acute renal disease and serves as a useful biomarker for renal proximal tubule injury [21,24]. Therefore, we evaluated the effect of PV on decreasing the renal damage caused by CP administration by measuring the relative renal expression of KIM1. The administration of CP caused a significant (*p* < 0.05) elevation in KIM1 expression compared to the three control groups. On the other hand, the treatment with PV 100 mg or 200 mg exhibited a significant (*p* < 0.05) lowered KIM1 when compared to the CP group (Figure 9H).

## 3. Discussion

In silico tools such as network pharmacology and molecular docking emerged as a reliable tool for identifying the interaction and correlation between different pathways so that we can gain new insights about unexplored mechanisms of actions of drugs [25]. In this study, the speculated network shed light on major pathways involved in the pharmacological action of PV. Through gene ontology (GO) analysis, a set of terms was enriched to describe certain biological processes (BP), molecular functions (MF), and cellular components (CC). For BP, inflammation-associated processes were the main enriched term, especially through inflammatory pathways and phosphorylation of a wide range of kinases, which would be explained in the context of MF, where MAPK activation and serine/threonine kinase activity were among the top enriched functions.

This agrees with previous reports indicating that MAPKs play a crucial role in the development of CP-induced toxicity [4,6]. Apparently, MAPK inhibition not only led to the downregulation of inflammation induced by CP but also reduced its anti-proliferative effect on the kidney by blocking the activation of Bax and preventing apoptotic cascades [26]. This was well demonstrated in our model, where downregulation of Bax and upregulation of Bcl2 in rats co-treated with PV and CP were significantly observed.

KEGG analysis emphasizes pathways that are usually activated in hypoxic environments and increased oxidative stress, such as the AGE-RAGE signaling pathway, which is usually activated in diabetes [27]. On the other hand, inhibition of RAGE has been recently reported as a promising approach to alleviate nephrotoxicity induced by CP [28]. Furthermore, PV was identified as a lead compound that could inhibit RAGE [29,30], which confirms the accuracy of our network pharmacology results. Other enriched terms of the KEGG are related to lipid and atherosclerosis, which could be explained in the context of previous studies showing that CP-induced lipid accumulation through the blocking of fatty acid oxidation leads to inflammation, cell injury, and, finally, the activation of apoptotic pathways [31]. Finally, the activation of inflammatory pathways such as IL-17 and TNF-α signaling pathways could be addressed in light of the enrichment of MAPK1 as the most significant target. Since MAPK1 activation occurs exclusively in the distal nephron, it plays an important role in distal nephron injury [32].

Also, MAPK1 inhibition by other natural products, such as galangin, was found to mitigate nephrotoxicity induced by CP [33]. Moreover, PV was reported to inhibit MAPK1 in different cell lines; such activity was attributed to its PDE inhibitory effect, which consequently prevented the AMP-dependent activation of ERK signaling [34,35]. Moreover, our results indicated the ability of PV to reverse the increased expression of MAPK1 induced by CP in a dose-dependent manner. Also, our molecular docking study suggested the ability of PV to bind effectively to its ATP active site. This could be supported by the observation by Aggarwal et al. that PV inhibits the phosphorylation of MAPK1 without affecting the non-phosphorylated MAPK1 [36].

The enriched molecular targets of papaverine (PV) significantly overlapped with pathways implicated in cisplatin (CP)-induced nephrotoxicity, validating the network predictions. Based on these insights, we selected key biomarkers related to mitochondrial dysfunction, oxidative stress, apoptosis, inflammation, and kidney injury to evaluate PV’s potential to mitigate these nephrotoxic effects at the cellular and molecular level in vitro and in vivo.

Specifically, we examined PV’s ability to prevent CP-mediated damage to renal epithelial cells and nuclear and mitochondrial DNA alongside modulation of antioxidant systems, apoptotic signaling, cytokine production, and histopathological damage that collectively mediate acute kidney injury [37].

In vitro, papaverine (PV) significantly reduced cisplatin (CP)-mediated toxicity in normal kidney cells without compromising efficacy against cancer cell lines. We confirmed this renoprotective effect in vivo, whereby PV alleviated CP-induced renal dysfunction, acute tubular damage, pathological lesions, inflammation, and apoptosis in rat models. This may be explained by PV’s inhibitory effects on phosphodiesterases (PDEs), which regulate intracellular cAMP and related DNA repair and cell differentiation cascades. Since renal inflammation can arise from CP-induced oxidative stress and apoptosis, PDE emergence as a nephrotoxic mediator further rationalizes PV’s protective effects, given precedents of PDE inhibition managing inflammatory diseases including COPD, IBD, psoriasis, and CNS inflammation [38,39]. It has been reported that inhibitors of PDE10 have potential therapeutic effects in several diseases, including neurological diseases [14] and cardiotoxicity [40].

While there are no previous reports addressing the ability of PV to protect renal cells, previous reports showed that it possesses a cytoprotective effect against cellular injury. For instance, it was found to protect against lipopolysaccharide (LPS) induced inflammation in BV2 microglial cells [41]. Also, it protects human cortical neurons against the toxicity of quinolinic acid due to the upregulation of the cAMP cascade, which subsequently decreases oxidative stress [42]. Hence, the antioxidant and anti-inflammatory effect of PV in vitro could explain the demonstrated protective effect of PV on CP cytotoxicity, which is our study.

CP-induced nephrotoxicity in experimental animals is characterized by variations in renal morphology, marked apoptosis of renal cells, and significant increases in serum urea and creatinine [43]. Urea and creatinine serum levels were markedly elevated upon CP administration. These changes were reversed by PV treatment. Additionally, histology examination of kidney tissues also confirmed that PV treatment could improve CP-mediated kidney pathological damage. Additionally, variations in KIM1 expression have been associated with the progression of CP-induced renal damage. Its level also alters earlier than any conventional biomarker of kidney damage [44]. Our study revealed that the treatment with PV significantly decreased the renal expression of KIM1 compared to the CP group. These findings could prove the nephroprotective role of PV treatment.

The pathogenesis of acute kidney disease includes unreasonable oxidative stress, renal tubular epithelial cell apoptosis, and inflammation, which eventually lead to the progression of chronic renal disease [45,46,47]. Elevated MDA induces over-release of reactive oxygen species (ROS), leading to oxidative stress that inhibits CAT, GPx, and SOD activities and promotes tissue damage [47,48,49]. In addition to ROS, nitrosative stress was also observed, which is characterized by an elevation in the iNOS production, resulting in an increase in nitric oxide that mainly contributes to CP-induced kidney injury and toxicity [50,51]. In consistence, we also found that PV increased the activities of CAT, GPX, and SOD and the expression of their genes (SOD3 and CAT), and decreased MDA level and the expression of the iNOS gene in the kidney. These data indicated that PV has antioxidant activities, which participate in alleviating CP-induced renal toxicity.

Oxidative stress also plays an important role in the activation of the NF-κB pathway and increasing the expressions of pro-inflammatory genes, such as TNFα and IL6. NF-κB is an important transcription factor regulating inflammatory responses in CP-induced renal damage [52]. Consequently, the release of inflammatory factors, including TNFα, increased upon NF-κB signaling activation mediated by renal damage [53]. Additionally, IL10 is a potent anti-inflammatory cytokine that exerts its action by binding at several sites in the inflammatory cascade. It suppresses neutrophil-mediated inflammation, tissue injury, and iNOS induction. IL10 was found to decrease renal damage in different models of renal damage, including transplantation of a marginal kidney, ischemia, and CP-mediated kidney injury in murine animals [54]. MCP1, a potent chemotactic factor for monocytes, was used as an early diagnostic marker in acute kidney injury in murine models [55]. Previous studies also illustrated that CP induces nephrotoxicity through the activation of the MAPK pathway, which leads to increased TNFα production [7]. Our study revealed that PV administration exhibited a significant decrease in TNFα, IL6, MCP1, and MAPK1 and a significant increase in IL10 gene expression compared to the CP group. These data inferred that PV treatment could significantly reduce CP-induced renal damage via its anti-inflammatory activities.

It has been reported that CP promotes the overproduction of ROS, which induces apoptosis [56,57]. Several apoptosis-associated proteins, including caspase family members and Bax, can promote apoptosis caused by CP nephrotoxicity. The translocation of Bcl2 family proteins is also associated with CP-mediated DNA renal damage [58]. Our findings revealed that the treatment with PV significantly decreased the CP-induced cellular apoptosis via down-regulating the renal gene expressions of Bax and upregulating the expression of the Bcl2 gene in renal tissues. These data implied that PV could improve CP-induced renal damage by regulating apoptotic responses.

## 4. Materials and Methods

### 4.1. Network Pharmacology and In Silico Prediction of PV Targets

PV 3D was retrieved from the PubChem database (https://pubchem.ncbi.nlm.nih.gov/) (accessed on 1 January 2024). Using Pharm Mapper (http://www.lilab-ecust.cn/pharmmapper/) (accessed on 1 January 2024). and Swiss target prediction server (http://www.swisstargetprediction.ch/) (accessed on 1 January 2024). we obtained 189 potential drug targets [59]. Using the gene cards database (https://www.genecards.org/) (accessed on 1 January 2024) we obtained 289 molecular targets related to nephrotoxicity. Common targets among the two sets were retrieved by Venny 2.0 online tool (https://bioinfogp.cnb.csic.es/tools/venny/) (accessed on 1 January 2024). Gene ontology and pathway analysis were performed as described previously [60] to highlight enriched genes responsible for biological processes, molecular functions, cellular processes, and the KEGG pathways related to nephrotoxicity. Molecular docking was applied to obtain insights about PV-MAPK1 interaction. PV 3D structure was retrieved from PDB using the code:4O6E, and the 3D structure of PV was retrieved from PubChem in SDF format. Leadit software 2.8 was used as previously reported [61,62,63] and it showed RMSD = 1.3Å after redocking the co-crystallized ligand indicating its validity.

### 4.2. Drugs, Chemicals, and Kits

PV powder was purchased from Sigma Chemical Co. (St. Louis, MO, USA, Cat # P3510), CP (50 mg/50 mL) from EIMC United Pharmaceuticals (Cairo, Egypt), both Dulbecco’s Modified Eagle’s medium (DMEM) and heat-inactivated fetal bovine (FBS) serum from GIBCO (Grand Island, NY, USA), MTT from Molecular Probes (Eugene, OR, USA), dimethyl sulfoxide (DMSO) from Sigma Aldrich (Burlington, MA, USA), carboxymethylcellulose (CMC) from El-Gomhouria Company (Cairo, Egypt), Trizol reagent from Invitrogen (Waltham, MA, USA), RevertAid H Minus Reverse from Thermo Scientific (Waltham, MA, USA), and SYBR Green 2XMaster Mix from QuantiTect (Quiagen, Hilden, Germany). All other used chemicals were of high quality.

### 4.3. Cytotoxicity by the MTT Assay

The normal kidney cell line (Vero) was bought from Vacsera (Giza, Egypt). To determine whether CP and/or PV had a cytotoxic impact on Vero cells, we utilized the MTT assay. Vero cells (10^4^ cells per well) were cultured in DMEM containing 10% FBS, and CP and/or PV were added at doses ranging from 0 to 100 μg/mL before the cells were grown for 24 h. MTT wone.as then added at a concentration of 12 mM (10 μL/well) with further incubation (37 °C/4 h). The formed purple crystals were dispersed in 100 μL DMSO for half an hour. IC_50_ was determined by measuring the optical density (570 nm) and then plotting the data on a sigmoidal curve in GraphPad Prism 7.

### 4.4. Experimental Design

**Figure 10:**
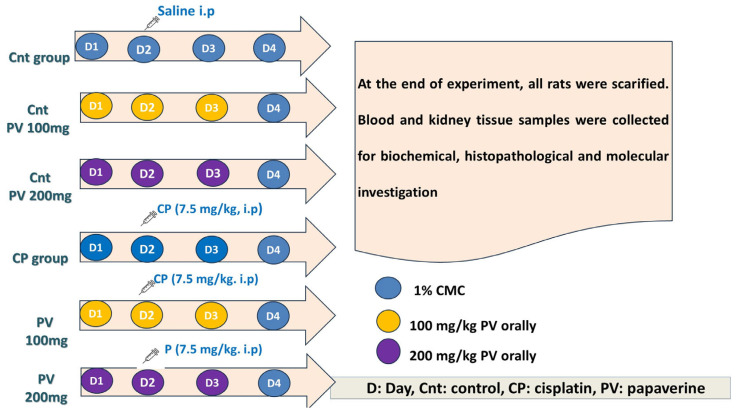
Schematic diagram of the experimental design. CP: cisplatin, PV: papaverine, and i.p: intraperitoneal injection.

This study was performed on 42 male, 6-week-old Swiss albino rats (100–150 g body weight). The ethical committee approval for all the experiment procedures (KFS-IACUC/141/2023) was obtained from KFS-IACUC ethical committee in Kafrelsheikh University, Egypt. The rats were given free access to food and water and were subjected to a constant temperature (21–25 °C) and a light/dark cycle of 12 h. Rats were acclimatized for seven days and randomly sorted into 6 groups (*n* = 7/group), as shown in Figure 10. Animals in the control (Cnt) group were intraperitoneally (i.p) injected with 0.9% saline once on Day 1 (D1) for 3 days (D1–D3). In the two PV control groups, animals were orally administrated PV at a concentration of 100 and 200 mg/kg in 1% CMC at Day 1 (D1) for 3 days (D1–D3) [64]. In the CP group, rats were i.p injected with a single dose of 7.5 mg/kg b wt CP dissolved in 0.9% saline on the second day (D2) of the study [65]. In the two PV co-treated groups, rats were co-treated with CP (as in the CP group) and either 100 mg/kg or 200 mg/kg PV in 1% CMC orally for 3 days (D2–D4).

### 4.5. Sampling

Three days after CP administration, all animals were fasted, and blood was withdrawn from the ocular venous plexus. Following centrifugation (3000 rp/5 min), serum was collected for creatinine and urea assessment. After rats were euthanized by exsanguination, their kidneys were taken out and cut in thirds: the first 1/3 was immersed in 10% formalin for histopathological examination, the second 1/3 was dipped in liquid nitrogen and then at −80 °C for qPCR, and the last 1/3 was homogenized in PBS (12,000× *g*/15 min/4 °C) to obtain supernatants for the biochemical analysis.

### 4.6. Kidney Function Tests

The serum creatinine and urea levels were determined using a colorimetric method according to Fossati et al. [66] and Patton and Crouch [67] methods, respectively, using commercially available kits (Bio-Med, Cairo, Egypt).

### 4.7. Biochemical Assays of Oxidative Stress

Malondialdehyde (MDA) content and catalase (CAT) activity in the kidney tissues were measured based on the methods reported by Utley et al. [68] and Aebi [69], respectively. Renal superoxide dismutase (SOD) and glutathione peroxidase (GPx) contents were also measured according to Nishikimi et al. [70] and Lawrence and Burk [71], respectively, using commercially available kits (Biodiagnostic, Cairo, Egypt) and as previously detailed [49,72].

### 4.8. Real-Time PCR

The qPCR was applied to determine the expression of Kim1, iNOs, the antioxidant genes (CAT and SOD3), inflammation-related genes (TNFα, MCP1, IL6, and IL10), and apoptosis-related genes (Bax and Bcl2) following the administration of CP and/or PV. Total RNA was extracted from kidney tissues of all groups using Trizol reagent, and cDNA was obtained following reverse transcription using a reverse transcription kit. The qPCR mixture containing cDNA, SYBR Green, and primers (Table 2) was operated with therrma1 cycles as previously detailed [73]. The fold changes of candidate genes were calculated by the Livak method (2^−∆∆Ct^) with the β actin as a housekeeping gene.

### 4.9. Histopathological Examination of Kidney Tissues

Paraffin-embedded kidney tissues were sectioned thinly (4 µm), stained with hematoxylin and eosin (H&E), and examined by a light microscope. The kidney injury score was established based on the following histopathological changes: (1) vacuolar degeneration of renal tubule cells; (2) nuclear pyknosis of renal tubule cells; (3) infiltration of inflammatory cells; (4) congestion of renal blood. These lesions were found in 10 non-overlapping areas selected at random and analyzed at 40× magnification. A score of 0 indicates no damage, 1 indicates 25% damage or less, 2 indicates 26% to 50%, 3 indicates 51% to 75%, and 4 indicates 76% or more [74].

### 4.10. Statistical Analysis

Statistical variations among different groups were detected by one-way ANOVA followed by the post hoc Tukey’s test as determined by GraphPad Prism version 8.0. Data were presented as mean ± SEM with significant results at *p* < 0.05.

## 5. Conclusions

PV could alleviate CP-induced renal toxicity by regulating oxidative stress, inflammation, and apoptosis. Network pharmacology analysis and molecular docking studies showed the complexity of the mechanism of action of PV, explaining its ability to modulate several pathways related to kidney injury. CP is widely used as an effective chemotherapeutic drug; still, its adverse effects substantially limit its use. Consequently, PV could be a possible therapeutic agent for the management of CP-caused renal injury. Although in vitro studies suggested that PV would not interfere with the therapeutic activity of CP as an anti-cancer agent, the therapeutic effect of combined treatment with PV and CP for solid tumor patients remains unknown and needs further investigation.

## Figures and Tables

**Table 1:** Gene distribution and correlation to KEGG enrichment, different biological processes (BP), and molecular functions (MF).

ID	Gene Symbol	Numbers Related KEGG	Numbers Related BP	Numbers Related MF
1.	* MAPK1 *	100/144	133/1331	7/92
2.	* MAPK8 *	70/144	90/1331	9/92
3.	* MAPK10 *	70/144	13/1331	6/92
4.	* TNF *	63/144	536/1331	5/92
5.	* MAPK14 *	60/144	151/1331	9/92
6.	* PRKCA *	53/144	98/1331	5/92
7.	* IL6 *	47/144	340/1331	6/92
8.	* BAX *	43/144	243/1331	5/92
9.	* BCL2 *	40/144	288/1331	6/92
10.	* CREBBP *	23/144	40/1331	1/92
11.	* JAK2 *	22/144	278/1331	11/92
12.	* IL10 *	20/144	258/1331	5/92
13.	* CDK2 *	17/144	38/1331	3/92
14.	* CCL2 *	16/144	129/1331	5/92
15.	* ICAM1 *	15/144	59/1331	1/92
16.	* HSP90AA1 *	14/144	107/1331	14/92
17.	* NOS2 *	12/144	84/1331	11/92
18.	* ATR *	6/144	56/1331	5/92
19.	* SELE *	6/144	25/1331	2/92
20.	* NQO1 *	4/144	59/1331	6/92
21.	* CYP1A1 *	3/144	75/1331	16/92
22.	* G6PD *	2/144	80/1331	2/92
23.	* S100A9 *	1/144	65/1331	10/92
24.	* ANXA5 *	0/144	5/1331	1/92
25.	* DPEP1 *	0/144	31/1331	4/92
26.	* ALAD *	0/144	45/1331	2/92

**Table 2:** Primers used for the qPCR.

Gene	Forward Primer (5′-----3′)	Reverse Primer (5′-----3′)
* MAPK1 *	AGGGCGATGTGACGTTT	CTGGCAGGGTGAAGTTGG
* iNOs *	CACCACCCTCCTTGTTCAAC	CAATCCACAACTCGCTCCAA
* SOD3 *	AAGGAGCAAGGTCGCTTACA	ACACATCAATCCCCAGCAGT
* CAT *	GAATGGCTATGGCTCACACA	CAAGTTTTTGATGCCCTGGT
* TNFα *	GCATGATCCGCGACGTGGAA	AGATCCATGCCGTTGGCCAG
* MCP1 *	TCGCTTCTGACACCATGCA	TGCTACAGGCAGCAAATGTGA
* IL6 *	TCCTACCCCAACTTCCAATGCTC	TTGGATGGTCTTGGTCCTTAGCC
* IL10 *	GTTGCCAAGCCTTGTCAGAAA	TTTCTGGGCCATGGTTCTCT
* Bax *	ACACCTGAGCTGACCTTG	AGCCCATGATGGTTCTGATC
* Bcl2 *	ATCGCTCTGTGGATGACTGAGTAC	AGAGACAGCCAGGAGAAATCAAAC
*KIM1*	TGGCACTGTGACATCCTCAGA	GCAACGGACATGCCAACATA
*β-actin*	AAGTCCCTCACCCTCCCAAAAG	AAGCAATGCTGTCACCTTCCC

## Data Availability

The data supporting the present findings are contained within the manuscript.
